# Supplementary material for: Light-Emitting Diodes Based on InGaN/GaN Nanowires on Microsphere-Lithography-Patterned Si Substrates
Source: Nanomaterials (Basel). 2022 Jun 10;12(12):1993. doi: 10.3390/nano12121993 (PMC9230727; doi:10.3390/nano12121993)
Supplement: Supplementary file 1 [file nanomaterials-12-01993-s001.zip › nanomaterials-1742117-supplementary.pdf]

Supplementary information

Light-Emitting Diodes Based on InGaN/GaN Nanowires on Microsphere-Lithography-Patterned Si Substrates

Liliia Dvoretckaia[1], Vladislav Gridchin[1,2], Alexey Mozharov[2], Alina Maksimova[1], Anna Dragunova[3], Ivan Melnichenko[3], Dmitry Mitin[4], Alexandr Vinogradov[4], Ivan Mukhin[1,4,5,*] and Georgy Cirlin[1,2]

1 Department of Physics, Alferov University, Khlopina 8/3, 194021 St. Petersburg, Russia; liliyabutler@gmail.com (D.L.N.); gridchinvo@yandex.ru (G.V.O.); deer.blackgreen@yandex.ru (M.A.A.); george.cirlin@mail.ru (C.G.E.)
2 Institute of Physics, Saint Petersburg State University, Universitetskaya Emb. 7/9, 199034 St. Petersburg, Russia; alex000090@gmail.com
3 Department of Physics, National Research University Higher School of Economics, Kantemirovskaya 3/1 A, 194100 St. Petersburg, Russia; anndra@list.ru (D.A.S.); imelnichenko@hse.ru (M.I.A.)
4 Department of Chemistry, ITMO University, Lomonosova 9, 197101 St. Petersburg, Russia; mitindm@mail.ru (M.D.M.); avv@scamt-itmo.ru (V.A.V.)
5 Higher School of Engineering Physics, Peter the Great St. Petersburg Polytechnic University, Polytechnicheskaya 29, 195251 St. Petersburg, Russia
* Correspondence: imukhin@yandex.ru

S1. Electron backscatter diffraction study

Scanning electron microscopy (SEM) of the synthesized arrays of InGaN/GaN NWs revealed the changing of NW facets during the growth from NW base to the top (see the insert in Figures 2 b and Figure S1). This can be governed by two factors: a rotation of the crystal lattice by 30 degrees or a change in the dominant facet. The inserts in Figure S1 show the electron backscatter diffraction patterns acquired in two different points depicted as (1) and (2). These points corresponded to different orientations of facets. The numbers on the inserts denoted the corresponding crystallographic planes. One can see that the presented electron diffraction patterns are the same for both points, proving the change in the dominant facet.

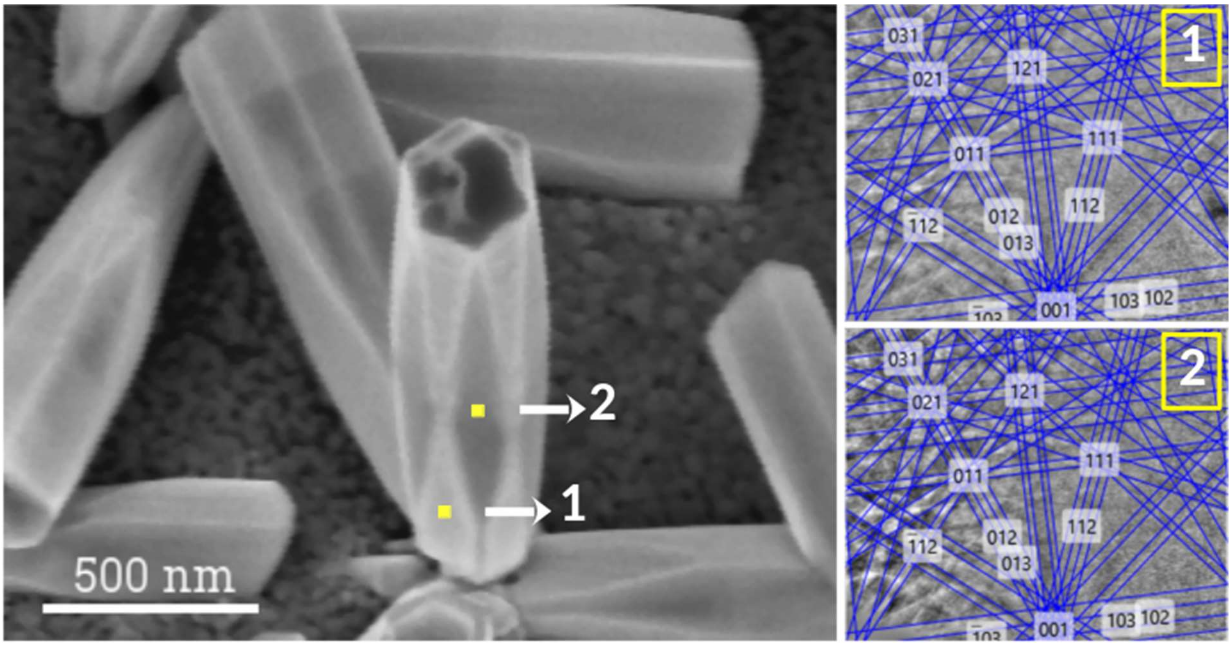

Figure S1. SEM image of the grown InGaN/GaN NWs. The inserts show the electron diffraction patterns acquired in point (1) and (2).
